# Cell Colonization Ability of a Commercialized Large Porous Alveolar Scaffold

**DOI:** 10.1155/2017/8949264

**Published:** 2017-12-13

**Authors:** S. Lemonnier, T. Bouderlique, S. Naili, H. Rouard, J. Courty, N. Chevallier, P. Albanese, T. Lemaire

**Affiliations:** ^1^Laboratoire Modélisation et Simulation Multi Echelle-Biomécanique (MSME), UMR 8208 CNRS, Université Paris-Est, 61 avenue du Général de Gaulle, 94010 Créteil, France; ^2^Laboratoire Croissance, Réparation et Régénération Tissulaires (CRRET), EA 4397, ERL 9215 CNRS, Université Paris-Est, 61 avenue du Général de Gaulle, 94010 Créteil, France; ^3^Laboratoire Bioingénierie Cellulaire, Tissulaire et Sanguine à Visée Thérapeutique, EA EFS 3952, Université Paris-Est, 51 avenue du Maréchal de Lattre de Tassigny, 94010 Créteil, France; ^4^Department of Laboratory Medicine (LABMED), H5, Division of Clinical Immunology, Karolinska Universitetssjukhuset, Huddinge, F9 14186, Stockholm, Sweden

## Abstract

The use of filling biomaterials or tissue-engineered large bone implant-coupling biocompatible materials and human bone marrow mesenchymal stromal cells seems to be a promising approach to treat critical-sized bone defects. However, the cellular seeding onto and into large porous scaffolds still remains challenging since this process highly depends on the porous microstructure. Indeed, the cells may mainly colonize the periphery of the scaffold, leaving its volume almost free of cells. In this study, we carry out an *in vitro* study to analyze the ability of a commercialized scaffold to be *in vivo* colonized by cells. We investigate the influence of various physical parameters on the seeding efficiency of a perfusion seeding protocol using large manufactured bone substitutes. The present study shows that the velocity of the perfusion fluid and the initial cell density seem to impact the seeding results and to have a negative effect on the cellular viability, whereas the duration of the fluid perfusion and the nature of the flow (steady versus pulsed) did not show any influence on either the fraction of seeded cells or the cellular viability rate. However, the cellular repartition after seeding remains highly heterogeneous.

## 1. Introduction

Critical-sized bone defects, as part of atrophic bone nonunions, require specific therapeutic protocols to restart the healing process and restore the mechanical continuity of the wounded bone [1]. Despite recent progress, the available treatments are still not satisfying since they involve long months of immobilization and multiple surgeries, do not guarantee a full recovery, and are often associated with important side effects [2–4]. To prevent the risks inherent to bone grafts (infection, complication at the donor site for autografts, and rejection for allografts), new synthetic biocompatible scaffolds have been developed to fill the bone defect and provide a mechanical support for bone reconstruction. These filling biomaterials are currently used for small bone defect reconstruction. However, for large implants, the cellular colonization of such scaffolds remains challenging in situ, due to the absence of chemical factors and preexisting cells usually initiating the migration of external cells towards the center of the lesion site [1, 5]. In this configuration, bone remodeling cannot take place in the volume of the scaffold, leading to its progressive weakening and then the fracture of 60% of such implants or grafts after 10 years [6].

The availability of biocompatible scaffolds homogeneously colonized by cells seems therefore to be a key parameter in the development of two therapeutic protocols dedicated to critical-sized boned effects: (i) the filling of the defect with a biocompatible material and (ii) the controlled *in vitro* development of tissue-engineered implants, coupling a biocompatible scaffold with cells and biochemical factors, that would then be implanted on the lesion site [7].

### 1.1. Filling Biomaterials

To address the downsides of autograft and allograft, companies worldwide have developed synthetic materials, the most widely used in bone defect treatment being calcium phosphates, calcium sulfates, and hydroxyapatite [8]. These bone graft substitutes, which can be pastes, aggregates, or porous blocks, provide osteoconductive scaffolding onto which new bone may grow. They can also serve as vehicles for osteoinductive and osteogenic substances.

### 1.2. Engineered Tissue Implants

This alternative method, although technically challenging, would ensure the filling of the defect with a living tissue able to produce the biochemical factors required to initiate the healing process.

In that prospect, several studies have been conducted during the past years to improve the cellular seeding and culture of tissue-engineered osteoarticular implants (cf.  Table 1). They typically involve mesenchymal stromal cells and/or cells from the osteoblastic and chondrocytic lineages, seeded on porous biocompatible scaffolds.

Although the chemical composition of the biomaterials used in these studies varies greatly, two types of scaffolds can be available such as medium-sized scaffolds (height ≤ 5 mm) with a porosity ≤ 80% and larger and highly porous ones with a porosity above 90%. The scaffolds of the first category show poor volumic colonization, leading to a heterogeneous tissue development during the *in vitro* culture phase [9–12]. On the other hand, the scaffolds of the second type seem to be more easily colonized but show poor mechanical properties [13–16].

### 1.3. Goal of this Study

In this paper, our goal is to analyze, thanks to an *in vitro* study, the ability of a commercialized scaffold to be *in vivo* occupied by cells. Using controlled fluid flow mimicking physiological *in vivo* conditions, we study the colonization efficiency of a commercialized large alveolar bone scaffold by mesenchymal stromal cells that have a prominent role in healing process. We thus mimic *in vivo* cell colonization by *in vitro* cell seeding. Our work is restricted to the early stage of the cellular seeding of the scaffold, that is to say the deposition of cells inside the volume thanks to fluidic stimulation. The idea here is to check how the perfusion conditions modify the competition between cellular advection with the fluid and cellular adhesion on the scaffold surface. In this stage, the scaffold microarchitecture plays a very important role. The following seeding process that consists in cellular migration is out of the scope of our analysis since it is often triggered thanks to chemoattractant biomolecules that are added in the seeding chamber.

Note that this study will also give indications on the possibility to optimize the perfusive transport of cells inside the volume of scaffolds to perform convenient tridimensional seeding before cultivating an implant. This study is thus restricted to analyze the immediate cellular seeding of a specified commercially used scaffold through different fluid perfusion protocols. Thus, our goal is only to check the possibility to carry cells inside the scaffold volume thanks to a perfusive flow. Each experiment being rather short (2-3 hours), the necessity to ensure oxygenation is useless. A perspective of this work would be naturally to improve this setup to analyze fluid-stimulated cell culture in a scaffold on a larger timescale. Indeed, it was shown that osteogenesis in cultured mesenchymal stromal cells can be modulated by scaffold and perfusion properties [17]. Indeed, the peculiar role of fluid stimulation on cellular migration and expansion [18, 19], on cellular mechanotransduction [20, 21], or on final implant quality [22] were put into relief.

Since we intend to provide reliable arguments on the practical application of large biomaterials in medical applications, we selected a *β*-TCP highly porous scaffold from the CERAVER company. This scaffold is already clinically used to repair large bone defects [23]. It is supposed to present a fully connected alveolar structure that may be seeded by cells under *in vivo* conditions. Our goal being to analyse the scaffold microstructure influence on the seeding ability, biomolecules that are often associated in clinical applications are not considered in this study.

Our experimental analysis draws its inspiration from the classical unidirectional perfusion seeding devices. The cylindrical scaffold is set inside a flow chamber where the cells are injected. Then, using a peristaltic pump, different mechanical stimulations are applied on the cells. Particularly, we focused on five parameters of the fluid flow which are the number of the injected cells *N*_cell_, the perfusion rate *V*_fl_, the cellular sedimentation time *T*_p_, the nature of the fluid flow (steady or pulsed), and the perfusion duration *t*_fl_. The impact of these parameters and the associated mechanical stimulations undergone by the cells during the seeding of the scaffold was evaluated analyzing the number of seeded cells, their localization within the scaffold, and their viability.

## 2. Materials and Methods

### 2.1. Cell Selection and Preparation

Human mesenchymal stromal cells (hMSC) were obtained from a unique bone marrow sample procured by the Etablissement Français du Sang, Hôpital Henri Mondor (Créteil, France), and expanded in *α*MEM (Gibco®, Life Technologies) supplemented with 10% foetal bovine serum (FBS, Gibco, Life Technologies) and 1% penicillin-streptomycin (100x, Gibco, Life Technologies). Culture medium was changed twice a week. For seeding tests, cells were washed with PBS, harvested using 0.05% Trypsin EDTA (Gibco, Life Technologies), and suspended in fresh culture medium at the density of 10^9^ cell/600 mL.

### 2.2. Scaffolds

**Figure 1:**
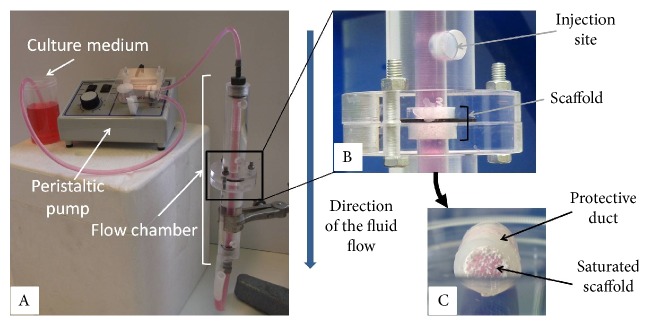
Experimental setup: A, general view of the perfusion chamber connected to the peristaltic pump; B, zoom in on the scaffold inside the flow chamber; C, view of the scaffold inside its silicon protective duct.

Alveolar ceramic cylinders (10 mm thick, diameter 8 mm, 100% *β*-TCP, porosity 75%, pore radius ~ 400 *μ*m, connection radius ~ 100 *μ*m) were procured by the CERAVER company. These scaffolds are already used for the filling of large bone defects. 24 hours prior to the experiments, they were placed in a protective silicon duct (see Figure 1, C) and immerged in *α*MEM (Gibco, Life Technologies).

### 2.3. Test Protocol

On the day of the test, scaffolds were placed in the center of a custom-designed flow chamber that ensures that the fluid flow goes entirely through the scaffold along its principal direction (see Figure 1, A). This chamber was saturated with culture medium and connected to a peristaltic pump.

Cells (*N*_cell_) were slowly injected in the saturated chamber above the scaffold (see Figure 1, B) using a 0.6 mm needle (NOELUS, TERUMO) and were allowed to sediment for a varying time *T*_p_. The pump was then activated to ensure the desired and constant fluid flow through the scaffold. Note that the silicon duct around the scaffold avoids any leaking near the chamber walls, forcing the flow to go through the porous structure. Finally, the system was allowed to rest for a minimum time of 150 min to ensure that the seeded cells had started to adhere to the scaffold. Since our analysis is restricted to check the possibility to carry cells inside the scaffold volume thanks to a perfusive flow and not to observe migration, each experiment remains rather short and the necessity to ensure oxygenation was useless.

### 2.4. Studied Parameters

Experimental tests were conducted varying 5 physical parameters (as shown in Table 2): the perfusion rate *V*_fl_, the initial cell number *N*_cell_, the sedimentation time *T*_p_, the nature of the fluid flow (steady or pulsed), and the perfusion distance *d*_fl_ *= V*_fl_ *× t*_fl_, where *t*_fl_ is the duration of the controlled perfusion. When not specified in a given test, the parameter value is set to the reference value (bold type in the table). These reference values have been chosen according to their ability to represent *in vivo* conditions and previously developed seeding protocols.

### 2.5. Seeded Cell Numeration

The number of seeded cells was evaluated adapting a protocol developed by [24]. PBS was slowly injected underneath the sample to collect the culture medium saturating that portion of the chamber. The sample was then washed with 4 different solutions at a constant perfusion rate of 5.77 × 10^−4^ m/s to collect the seeded cells. First, PBS was perfused for 15 min, followed by 0.5% mass type I collagenase (C-0130, Sigma®) for 30 min and 0.05% Trypsin EDTA for 10 min. Finally, PBS was applied once again for 20 min. Cells contained in each collected solution were then counted using a 0.2% Trypan Blue solution (Milerium, VWR) to evaluate their viability rate.

### 2.6. Data Analysis

The analysis of the variance (ANOVA) technique was used to study the significance of the influence of the five perfusion parameters of Table 2 on the cell viability rate and the quantity of seeded cells. Note that for each studied perfusion parameter, each experiment was reproduced (~3–5 times) to have a convenient statistical representation of the results.

### 2.7. Histology

For each perfusion condition, samples were extracted from the flow chamber and dehydrated through 9 successive baths with various ethanol solutions (70%, 80%, 90%, 95%, and 100% (three times)) and twice with xylene. They were then included in a poly(methyl methacrylate) (PMMA) matrix and cut along their principal direction (8 cuts per sample). Then, microscope observations were performed to analyze the cellular distribution within the porous structure.

## 3. Results

### 3.1. Initial Cell Number and Nature of the Flow

**Figure 2:**
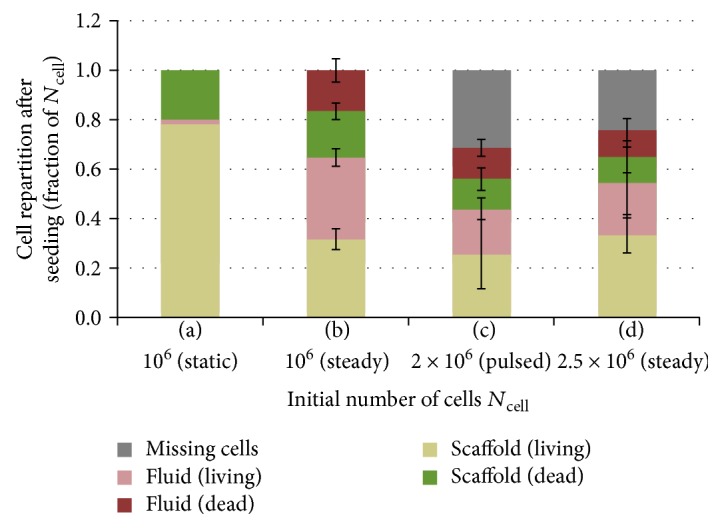
Influence of the initial cell number *N*_cell_ and the nature of the flow on the seeding efficiency.

The first set of tests focuses on the influence of the initial cell number *N*_cell_ and the nature of the flow on the seeding results. In Figure 2, we compare three different initial cell densities (samples (b) through (d)) submitted either to a steady fluid flow (*V*_fl_ = 9.7 × 10^−4^ m/s, *T*_p_ = 30 min, *d*_fl_ = 1.8 cm) or to a pulsed one described in Table 3.

Hence, the total perfusion distance for the samples submitted to the pulsed flow is 1.8 cm, at the average fluid velocity *V*_fl_ of 9.7 × 10^−4^ m/s. We also realized a static test (sample (a)) as a comparison tool already calibrated in a previously published study [25], using the exact same protocol without activating the peristaltic pump.

According to Figure 2, it is first noticed that 80% of the injected cells are seeded on the scaffold with the static protocol, whereas this rate is below 40% when a fluid flow is applied, regardless of the initial cell density *N*_cell_. Moreover, for sample (a), only a very small fraction of the initial cells is collected below the sample. On the other hand, when applying a controlled fluid perfusion, a large part of the injected cells go through the entire scaffold, which is encouraging to achieve a volumic cellular colonization.

The cellular viability rate after seeding is lower when a fluid flow is applied compared to the static protocol. This viability drop increases with the initial cell number *N*_cell_, although this trend is not statistically significant. Besides, the variability of the results seems more important at high initial cell densities (samples (c) and (d)). These two values of *N*_cell_ are also related with a missing fraction of the initial cells after the seeding test. We assumed that the missing cells had been destroyed throughout the process and could therefore be considered as additional dead cells located in the fluid.

Finally, changing the nature of the flow does not seem to have any impact on the cellular viability rate (samples (c) and (d)). Therefore, the other sets of experiments have been conducted using a steady fluid flow. At the early stage of the seeding, the role of the fluid seems thus mainly to carry cells until they cross the scaffold surface. Note that the type of the flow (pulsed or not) is known to have strong influence during the following culture stages [26].

### 3.2. Sedimentation Time

As they enter the flow chamber, the injected cells have already undergone various physical and chemical stresses (enzymatic actions, centrifugation, manipulation at room temperature, and injection). We suggested that a sedimentation time between the injection and the beginning of the fluid perfusion could then limit this stress accumulation, allowing the cells to rest before going through the scaffold. The results of the tests conducted varying parameter *T*_p_, and keeping the other parameters at their reference values, are presented in Table 2.

**Figure 3:**
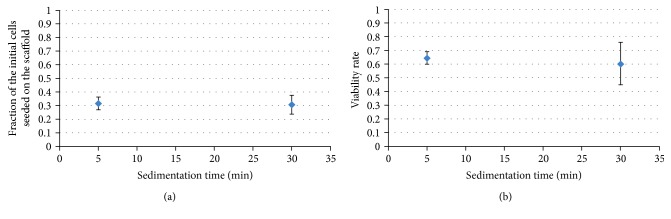
Influence of the sedimentation time *T*_p_ on the seeding results.

No change in the number of seeded cells (Figure 3(a)) nor in the viability rate (Figure 3(b)) were observed. Therefore, this parameter does not impact the perfusive seeding process.

### 3.3. Perfusion Distance

Next, we hypothesized that the variation of the perfusion distance *d*_fl_, achieved by varying the duration *t*_fl_ of the fluid perfusion, may highlight the existence of an optimal perfusion time allowing the cells to colonize the entire scaffold without being collected in the fluid underneath it. In addition, by varying the perfusion distance, we modify the duration of the mechanical solicitation applied to the cells, which could be a cause for the low viability rate observed after the seeding protocol.

**Figure 4:**
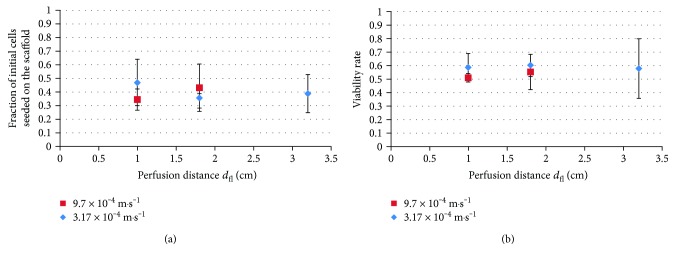
Influence of the perfusion distance *d*_fl_ on the seeding results.

The results presented in Figure 4 correspond to the seeding tests conducted varying the perfusion distance *d*_fl_ using two different perfusion velocities and keeping the parameters *N*_cell_ and *T*_p_ at their reference values (cf. Table 2).

No clear influence of this parameter can be observed on both the fraction of seeded cells and the viability rate for the considered range of perfusion velocity. This seems to indicate that the perfusion distance *d*_fl_ does not impact the seeding results either and could presume inadequate cellular penetration into the scaffold, which will be confirmed by histological observations.

### 3.4. Perfusion Velocity

**Figure 5:**
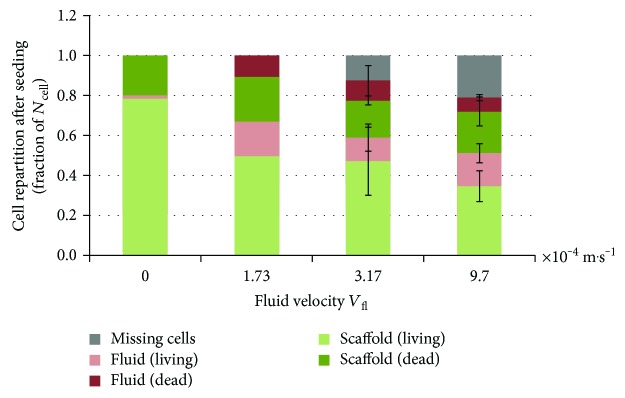
Influence of the fluid velocity *V*_fl_ on the seeding results. Tests with *V*_fl_ = 3.17 × 10^−4^ m/s and *V*_fl_ = 9.7 × 10^−4^ m/s have been conducted 3 times, whereas the static test and the test with *V*_fl_ = 1.73 × 10^−4^ m/s have only been conducted once as comparison tools.

Finally, we conducted a set of tests varying the fluid velocity *V*_fl_ and keeping the other parameters at their reference value (cf. Table 2). The results of these experiments are presented in Figure 5.

The fraction of seeded cells and the cell viability rate both decrease as the fluid velocity *V*_fl_ increases, although this trend is not statistically significant (mainly due to the high variability of the experimental data). In addition, a portion of the initial cells is missing for the two highest values of *V*_fl_.

### 3.5. Histological Observations

**Figure 6:**
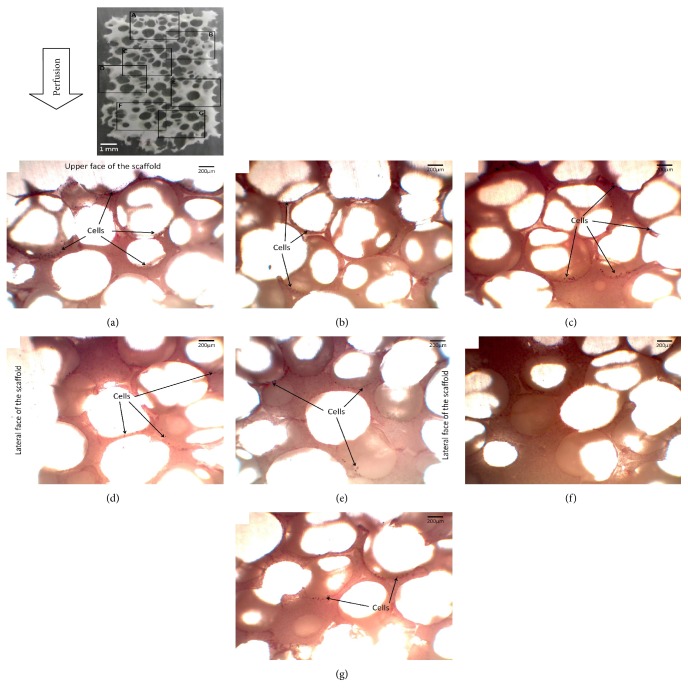
Histological slice of a scaffold seeded under controlled fluid perfusion.

Histological analysis of the seeded samples (8 slices per sample) has been performed to get qualitative information on the impact of the perfusion flow and the scaffold structure on cellular repartition after the different seeding protocols (two samples per protocol).  Figure 6, which corresponds to the reference case, roughly gives similar results as other seeding condition trials. The cells have been injected above the upper face of the scaffold. The sample was stained with Stevenel blue and van Gieson picrofuchsin. Observations were then conducted using an optic microscope.

This histological analysis indicates that the perfusion conditions do not strongly impact the cellular repartition within the porous structure. This repartition consists in an important cell layer on the pores located along the upper face of the scaffold (box A in Figure 6), and a rapidly decreasing cell density as we progress towards its lower face, up to macroscopic areas almost free of cells at the bottom of the scaffold (boxes F and G in Figure 6).

**Figure 7:**
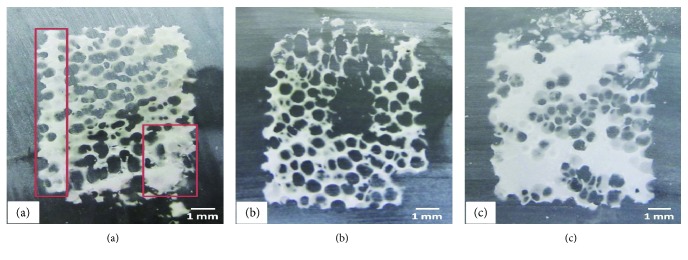
Different types of scaffold macroscopic structures.

Moreover, through this microscope analysis of several porous samples, we observed important variations in the porous structure of the scaffolds (Figure 7), with three different types of irregularities: macroscopic lacunae (Figure 7(b)) and accumulation of solid matrix in the volume of the scaffold (Figure 7(c)) or along its edges (red boxes, Figure 7(a)).

These irregularities could have a major impact on the seeding results, because they interfere greatly with the cell progression inside the scaffold. Besides, they lead to the formation of areas inaccessible to the cells, and could strongly impact the mechanical properties of the final implant.

## 4. Discussion

### 4.1. Influence of the Flow on the Seeding Results

According to the tests results, the static protocol seems to allow the seeding of a larger fraction of the injected cells onto the scaffold than the dynamic perfusion. However, it has been observed in previous studies [27, 28] that when using a static protocol, cells mainly stay above the sample, leaving the center of the scaffold almost free of cells. The very small fraction of the initial cells collected below the sample tends to confirm this analysis.

We show that the cellular viability was decreased with the level of perfusion, suggesting that the mechanical solicitations applied to the cells lead to permanent damages. We therefore proposed varying different physical parameters in order to identify the solicitation that could cause such damages.

According to our results, the sedimentation time *T*_p_, the type of the fluid solicitation, and the perfusion distance *d*_fl_ do not seem to be responsible for the low cell viability. On the other hand, the fluid velocity *V*_fl_ seems to have a negative impact on both the number of seeded cells and their viability. These results are rather surprising, considering that the velocities used in the present work have been found in previous studies to be not deleterious for the cells [29]. This discrepancy could be due to the different cell type used in our study. It is well known that viability of adherent cells is linked to their capability to adhere and spread onto a matrix. In this context, it could be to coat scaffold with molecules derived from an extracellular matrix compound that would optimize MSC adhesion.

### 4.2. Weak Ability of the Cells to Penetrate the Volume of the Bone Graft

It appears clearly that the cellular penetration within the 3D porous structure of this commercially used alveolar bone graft remains limited. Investigation of various perfusion parameters resulted in limited improvements in terms of 3D seeding. This suggests that, when filling a bone defect with such an alveolar ceramic graft, the cellular colonization of the porous volume is only efficient at the periphery of the scaffold. Thus, when comparing the cellular colonization versus optimal mechanical resistance, the use of less porous scaffolds may be of interest for *in vivo* bone repair applications.

This inability for cells to colonize the core of large biomaterials observed through our results is coherent with recently published studies. Indeed, scaffold with alveolar structure seeded with this type of tests are usually 5 mm in height at most (cf.  Table 1), and the cellular penetration in the scaffold rarely exceeds 2-3 mm.

A possibility to improve this limited efficiency would consist in using biomolecules that improve cell migration and tissue development. Moreover, the possibility to functionalize scaffold to provide more cell binding sites is another valuable avenue of research.

The alveolar structure could be partially responsible for these nonoptimal cell colonization results: although it provides a connected porosity and sufficient mechanical properties to the scaffolds, this structure includes brutal narrowing of the section of the fluid domain, leading to an early contact between the cells and the scaffold walls, thus promoting an early cellular adhesion. To enhance the cellular penetration in the scaffold without decreasing its mechanical properties, it could be interesting to optimize the shape and the size of the pores. Indeed, [10] have shown that the use of a regular porous structure leads to a more homogeneous cellular repartition after seeding in comparison with alveolar scaffolds. In addition, the use of a regular structure helps prevent important local variations of the mechanical solicitations (and of the shear stress in particular) that could cause cell damages in the concerned areas.

Finally, the shape of the porous domain and the cellular activity both evolve after the cellular seeding, during the *in vitro* culture phase. An optimized porous structure should then take into account this evolution and enhance an adequate fluid perfusion during this development phase.

The setup of an in silico study accounting for the main phenomenon controlling these phases seems therefore to be an appealing solution. Indeed, it would allow to identify the structure parameters that require optimization faster and more precisely than a solely experimental method [29].

## 5. Conclusions

According to the present study, the evaluated commercialized porous scaffold does not seem to be well adapted for homogeneous volumetric cell colonization. Indeed, although the cells are able to go entirely through the scaffold, the seeded cell rate after perfusion remains below 40% of the injected cells. In addition, the cell viability decreases rapidly, even under perfusion velocities that have been shown to be harmless to the cells in previous studies [28]. Moreover, histological observations have shown that a large majority of the seeded cells was located along the outer upper face of the scaffold (i.e., on the first face of the scaffold encountered by the cells), which seems to indicate that the alveolar structure does not promote volumetric penetration into the scaffolds.

These observations are coherent with various seeding studies available in the literature. Indeed, due to seeding limitations, scaffolds with similar structures rarely exceed 5 mm in depth, way below the size of clinical interest. Optimizing the porous structure to facilitate cellular and chemical transport through the scaffold might then be a promising way to improve the seeding and the *in vitro* development of tissue-engineered bone implants that meet this clinical size.

In addition to further experimental tests to understand these paradoxical results, it would therefore be interesting to conduct an in silico study to reveal the structural key parameters for the promotion of a homogeneous cell seeding in large bone scaffolds [30].

## Figures and Tables

**Table 1:** Various scaffolds and protocols used for the *in vitro* development of tissue-engineered bone scaffolds.

Ref.	Cell	Scaffold	Protocol	Observation
[14]	Rat MSC	60% HAP, 40% *β*-TCP Porosity 90% Cylinders (*h* = 8 mm, *D* = 8 mm)	Seeding: suction Culture: static versus fluid flow	Poor mechanical properties
[11]	Human MSC	HAP Porosity 80% Cylinders (*h* = 4 mm, *D* = 8 mm)	Seeding: static versus fluid flow Culture: static	Homogeneity (i) After 3 days ≤ 40% (ii) After 14 days ≤ 70%
[16]	Fluorescent particles	PCL Porosity 90% Parallelepipeds (*h* = 6 mm, *S* = 2 × 5 mm)	Seeding: static with acoustic waves	Homogeneous repartition in the first 3 mm, then gradient of particle concentration
[12]	Ovine MSC	*β*-TCP porosity not given Tubes (*h* = 30 mm, *D*_i_ = 3 mm, *D*_e_ = 14 mm)	Seeding: suction Culture: fluid flow	Gradient of cell concentration from the center towards the outside of the tube
[13]	MC3T3^1^	Polystyrene foam Porosity 95% Cylinders (*h* = 3 mm, *D* = 8 mm)	Seeding: static versus fluid flow	Few cells actually seeded on the scaffold Homogeneity: 40% (static) to 80% (fluid flow)
[15]	MG63^2^	PLA Porosity 95.7% Cylinders (*h* = 12 mm, *D* = 6 mm)	Seeding: fluid flow	Homogeneity ≤ 50% Poor mechanical properties
[9]	MC3T3	HAP Macroscopic canals Cylinders (*h* = 4 mm, *D* = 5 mm)	Culture: static versus fluid flow	Static: peripheral cellular colonization only Fluid flow: volumic colonization
[10]	Immortalized MSC	PDLLA-dimethacrylate Porosity 67% Cylinders (*h* = 5 mm, *D* = 8 mm)	Culture: static versus fluid flow	Comparison between alveolar and gyroïd structures: better cell homogeneity for the latter

A more detailed review can be found in [31]. *h*: height; *D*: diameter; *D*_i_: internal diameter; *D*_e_: external diameter; *S*: section. ^1^MC3T3: immortalized mouse osteoblastic precursors; ^2^MG63: human osteoblastic cells from an osteosarcoma.

**Table 2:** Studied parameters and their ranges of values.

Param.	Values (reference)	Observations
*V*_fl_	{0–3.17–**9.7**} × 10^−4^ m/s	Adapted from [20]
*N*_cell_	{**1**–2–2.5} × 10^6^ cells	10^6^ cells correspond to [18]
*T*_p_	{0–5–**30**} min	
*d*_fl_	{1–**1.8**–3.2} × 10^−2^ m	
Flow	{Steady, pulsed}	

The bold typeface corresponds to the reference values.

**Table 3:** Composition of the pulsed flow.

Time (s)	Cumulative perfusion distance (mm)
*t* = 0	0 (end of injection)
*t* = 60	2.08
*t* = 120	4.15
*t* = 300	6.92
*t* = 900	11.08
*t* = 1800	18

## Notations

*d*_fl_: Perfusion distance: *d*_fl_ *= V*_fl_ × *t*_fl_ (m)
*N*_cell_: Initial cell number (−)
*t*_fl_: Duration of the controlled perfusion (s)
*T*_p_: Sedimentation time (s)
*V*_fl_: Perfusion rate (m/s).

## Abbreviations

EDTA: Ethylenediaminetetraacetic acid
FBS: Foetal bovine serum
HAP: Hydroxyapatite
hMSC: Human mesenchymal stromal cells
MEM: Minimum essential medium
PBS: Phosphate buffer saline
PCL: Polycaprolactone
PDLLA: Poly-D,L-lactic acid
PLA: Polylactic acid
PMMA: Poly(methyl methacrylate)
TCP: Tricalcium phosphate.
